# Do teacher-training college students become more engaged in their studies because of commitment? The mediating role of self-control and the moderating role of core self-evaluation

**DOI:** 10.3389/fpsyg.2025.1569871

**Published:** 2025-07-22

**Authors:** Chen Li

**Affiliations:** School of Education, Taiyuan Normal University, Jinzhong, China

**Keywords:** learning engagement, professional commitment, self-control, core self-evaluation, teacher education, college students

## Abstract

**Objectives:**

As prospective educators, teacher candidates’ learning engagement and development require significant attention. This study aims to investigate the mechanism of the role between professional commitment and learning engagement of teacher-training college students.

**Methods:**

A questionnaire survey was conducted with 846 randomly sampled teacher-training college students using four validated scales: the *College Student Professional Commitment Scale*, the *Self-Control Dual-System Scale*, the *Core Self-Esteem Scale*, and the *Learning Engagement Scale*. The study employed descriptive statistics and structural equation modeling to validate the proposed measurement model and analyze the interconnections between the variables under investigation.

**Results:**

The results showed that (1) Different dimensions of professional commitment have significant effects on self-control; (2) Both the impulse and control systems of self-control significantly affect learning engagement; (3) The impulsive system and control system of self-control mediate the different dimensions of professional commitment; (4) The effect of the impulse system and control system of self-control on learning engagement is moderated by core self-evaluation.

**Conclusion:**

The study’s results reveal the mechanism of different dimensions of professional commitment’s influence on learning engagement, particularly the roles of self-control (including both impulsive and control systems) and the core self-evaluation. These findings provided valuable insights for designing intervention to enhance learning engagement among teacher education students.

## 1 Introduction

Students enrolled in teacher-training programs at universities and colleges are preparing to become educators across all levels of schooling. As future teachers, their mission is to contribute to the advancement of education, which is crucial for the sustainable development of the country.

In light of the societal demand for a top-tier education system and the cultivation of outstanding individuals, college students focusing on teacher training are essential to the creation of such a system. They embody the future of education and are instrumental in propelling educational advancement and elevating the standard of education.

However, the academic progress of teacher-training college students is directly linked to their potential as future educators. Their level of engagement in learning is a crucial indicator of their learning quality. It also serves as a significant predictor of their academic success, employment prospects, and job performance. At the same time, the extent to which students are engaged in their learning significantly impacts their own development.

Therefore, it is very important to focus on the learning engagement of teacher education college students and the factors that influence it. This attention is essential to improve the quality of teacher education college students themselves and to become a qualified and excellent educator.

## 2 Literature review

### 2.1 Professional commitment

Professional commitment is seen as an extension of organizational commitment and career commitment in the professional field (Zhang, 2022). It refers to an individual’s identification with the profession they are in, their willingness to put in efforts to achieve professional goals, and their emotional expression of love and pride (Morrow and Wirth, 1989). Professional commitment adds the ideal commitment dimension to the three-dimensional structural model of career commitment, which includes affective, continuing, and normative commitment (Allen and Meyer, 1990; Wang et al., 2021). Essentially, affective, continuing, normative, and ideal commitment formulate a four-dimensional theoretical model of professional commitment (Lian et al., 2005; Wang et al., 2021).

Affective commitment reflects a person’s deep emotional attachment and strong willingness to dedicate themselves to a career for the long term, working hard for it. Normative commitment refers to the sense of responsibility and obligation that individuals have for a specific occupation, which they consider a norm or standard that should be followed in order to participate in that occupation. Continuity commitment involves the individual’s clear perception of the costs of leaving a career, including possible financial loss, interruption of career progression, and impact on personal reputation. The ideal commitment reflects the strong expectations of college students regarding their majors. They firmly believe that by studying their chosen major, they can fully utilize their strengths and potential, and ultimately achieve their personal goals and aspirations. This commitment is not just a passion for their chosen profession, but also a steadfast pursuit of self-worth and future development (Lian et al., 2006; Wang et al., 2021).

### 2.2 Self-control

Self-control pertains to an individual’s capacity to adjust their behavior, restrain impulses and desires, and manage emotions to align with core values or achieve long-term goals (Baumeister et al., 2007). Based on current research, self-control can be considered a moral factor to some extent (Baumeister, 2012; Gai and Bhattacharjee, 2022; Mestvirishvili et al., 2023). Individuals are influenced by social expectations and make constructive adjustments to irrational perceptions and behaviors to achieve their long-term goals (Gai and Bhattacharjee, 2022).

The dual-systems model of self-control suggests that self-control consists of an impulsive system and a control system (Hofmann et al., 2009). According to this model, an individual’s control and impulsive traits should be considered to comprehensively measure self-control (Liu et al., 2020; Goschke and Job, 2023). The impulse system refers to the immediate behavioral impulses that naturally arise when an individual encounters a temptation. On the other hand, the control system represents the ability to resist and regulate such impulses that an individual demonstrates when faced with temptation (Gao et al., 2021).

Self-determination theory (SDT) suggests that human behavior is driven by voluntary and self-determined motives, which serve as the foundation and motivation for our actions. According to this theory, individuals are more likely to behave in a self-determined manner when they are in an environment that promotes autonomy and provides support. This self-determined behavior not only leads to increased engagement but also contributes to better performance (Deci and Ryan, 2000).

Furthermore, the dual-system model aligns with broader cognitive theories, particularly Kahneman (2011) distinction between System 1 characterized by automatic and intuitive processing and System 2 involving deliberative and effortful processing. Compared with the dual-systems model of self-control, the impulsive system parallels System 1 as it rapid, affect-driven responses to academic temptations such as procrastination. The control system reflects System 2 through its involvement in conscious regulation to align behavior with long-term goals including sustained study. This integration underscores how teacher trainees’ self-control dynamics operate within universal cognitive architectures while being shaped by profession-specific commitments as demonstrate (Hofmann et al., 2009).

Professional commitment, as a key indicator of college students’ learning attitudes, reflects the deep identification of students with their majors and their determination to make positive efforts toward this. It is a kind of self-control and decision-making by college students who, out of their love and recognition for their majors, are willing to invest their time and energy in in-depth study, exploration, and practice in the hope of achieving excellence and success in their fields of study. Previous research has commonly treated professional commitment as a unified construct. However, the specific ways in which its distinct dimensions, affective commitment, continuance commitment, normative commitment, and ideal commitment, may influence other variables have not been sufficiently examined. This gap is precisely the focus of our study.

Based on this premise, the current study hypothesized that all four dimensions of professional commitment (affective commitment, continuance commitment, normative commitment, and ideal commitment) would influence both the impulsive and control systems of self-control (H1–H8).

### 2.3 The mediating role of the dual system of self-control in professional commitment and learning engagement

Learning engagement is defined as the process of learning in which an individual shows a high level of energy, excellent mental toughness, a deep understanding of the meaning of learning, a passion for learning, and complete immersion in self-learning (Schaufeli et al., 2002). Specifically, it is the quality of the behavioral engagement and the emotional experience that students undergo when they consistently participate in learning activities. This engagement involves not only active participation in actions but also emotional commitment to learning. According to Hernandez and Garcia’s (2024) research, vigor, dedication, and absorption are considered the three dimensions of learning engagement. Prior research has thoroughly explored the various factors that influence engagement, such as motivational beliefs, achievement goals, academic procrastination, academic efficacy, enthusiasm for learning, and negative emotions. These factors not only independently impact engagement but can also interact and collaborate to shape the level of learner engagement (Dang et al., 2023).

The level of professional commitment has a significant impact on learning engagement. For example, research has shown that emotional and normative commitment in the workplace positively affects learning engagement (Yan et al., 2023; Ying et al., 2023). These factors also mediate the influence on learning engagement in relation to social support, family socioeconomic status, and the sense of belonging at home and school (Li and Shi, 2021; Dang et al., 2023). As a special group of college students, teacher trainees have a unique level of professional recognition, a strong sense of mission, and a heightened sense of responsibility that sets them apart from other types of college students. This is a topic that deserves to be explored.

According to the dual-system model of self-control and social cognitive theory, there is a close interaction between an individual’s behavior, cognition, and the environment. An individual’s cognition significantly influences their behavior. In other words, an individual’s way of thinking, conceptualizing, and understanding guides and shapes their outward actions (Bandura, 1986). Thus, a professional commitment, as perceived in the program of study, may influence students’ level of self-control. Meanwhile, it has been proved that the level of students’ learning engagement is affected by the level of self-control. When students’ self-control increases, their learning engagement also increases (Zhu et al., 2018; Wei, 2023). Additionally, self-control has a predictive effect on students’ learning engagement (Gao et al., 2020).

As a result, the four dimensions of professional commitment—namely affective commitment, continuance commitment, normative commitment, and ideal commitment—may influence teacher educators’ learning engagement through the two systems of self-control.

Based on this, the present study hypothesized that both self-control systems affect learning engagement (H9, H10); and that the dual system of self-control mediates the relationship between dimensions of professional commitment and learning engagement (H11, H12).

### 2.4 The moderating role of core self-evaluation

Core self-evaluation (CSE) was introduced by Judge in his research on personality tendencies for job satisfaction. He defined it as the most fundamental assessment an individual holds of their own abilities and values (Judge et al., 1997). Subsequently, numerous research studies have been conducted by scholars in various fields on core self-evaluation. Research has shown that a person’s academic progress is strongly connected to their core self-evaluation (Li et al., 2024; Zhang et al., 2024). Individuals with high levels of core self-evaluation are better equipped to handle different academic pressures and are less likely to experience issues such as anorexia, academic procrastination, and other factors that can impact their engagement with learning (Jung, 2021).

The conservation of resources theory’s results showed that coping strategies moderated the relationship between demand and inputs (Hobfoll and Stevan, 1989; Alarcon et al., 2011). Thus, as a positive psychological trait, core self-evaluation has the potential to have a protective effect on learning engagement by moderating the effects of some of the influences (e.g., self-control) on learning engagement. Thus, as a positive psychological trait, core self-evaluation has the potential to have a protective effect on learning engagement by moderating the effects of some of the influences (e.g., self-control) on learning engagement.

Based on this, this study hypothesized that core self-evaluation plays a moderating role between self-control and learning engagement (H13).

In summary, this study aims to investigate how the professional commitment of teacher educators influences their engagement in learning. It also examines the role of the dual-system model of self-control and the moderating role of core self-evaluation in this process. The goal is to determine whether teacher trainees with stronger educational ideals are more engaged in their studies due to their professional commitment and to understand how professional commitment impacts the learning engagement of teacher trainees.

**Figure 1:**
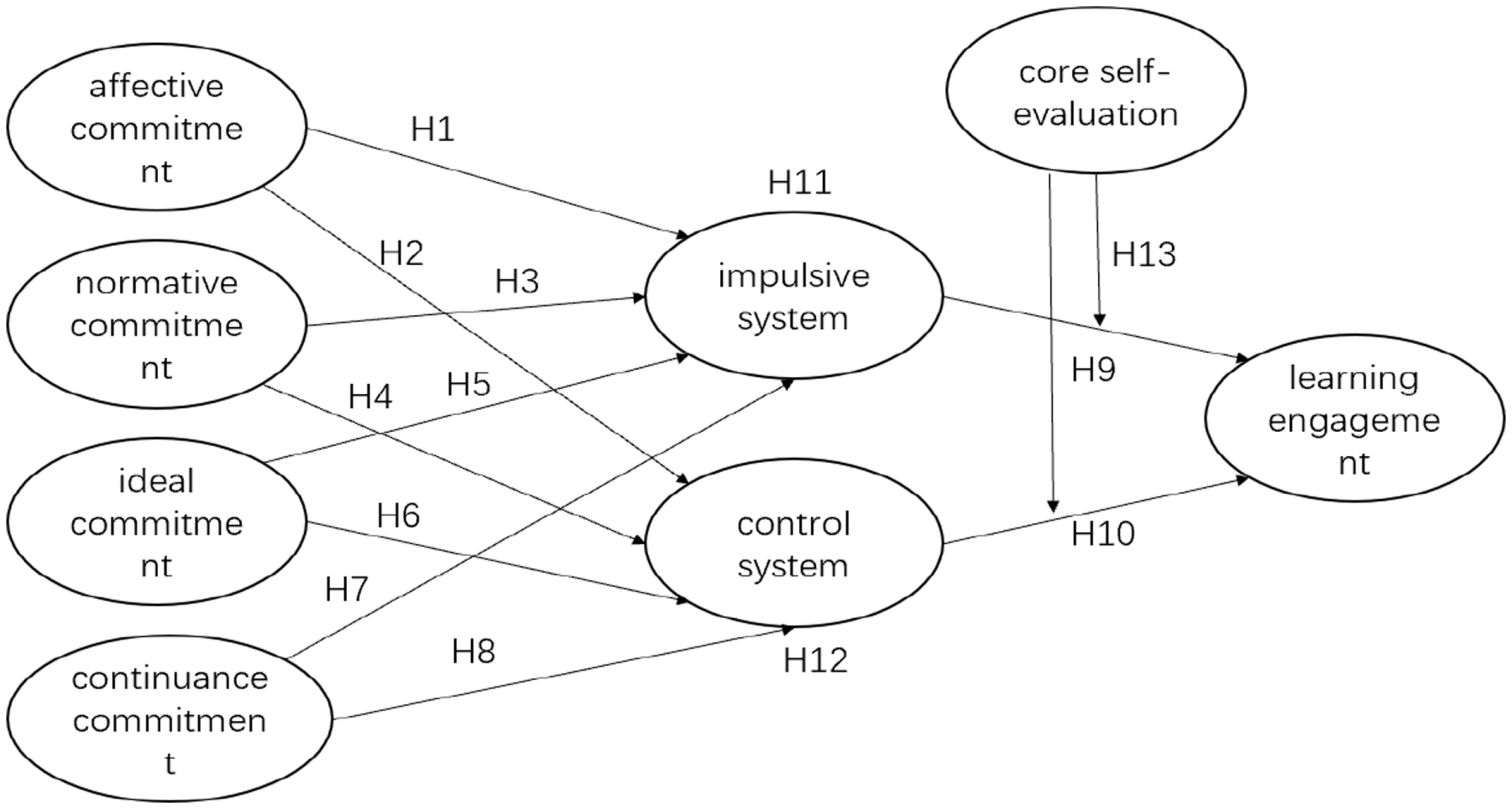
The hypothesized model of the four dimensions of professional commitment influencing learning engagement.

The study also aims to establish a theoretical foundation for improving teacher trainees’ learning engagement through the proposed model (Figure 1). The hypotheses are summarized as follows:

*H1–H8*: All four dimensions of professional commitment (affective commitment, continuance commitment, normative commitment, and ideal commitment) would influence both the impulsive and control systems of self-control.

*H9–H10*: Both self-control systems affect learning engagement.

*H11–H12*: The dual system of self-control mediates the relationship between dimensions of professional commitment and learning engagement.

*H13*: Core self-evaluation plays a moderating role between self-control and learning engagement.

## 3 Research methods

This study surveyed 846 randomly selected teacher-training students using four validated scales. Data were analyzed through descriptive statistics and structural equation modeling to validate the measurement model and examine variable relationships.

### 3.1 Participants and procedure

The study collected 894 questionnaires from teacher training colleges and universities using random sampling. After excluding 48 incomplete responses (those with less than one-third completion or patterned answers), the final sample consisted of 846 participants. Missing data were handled using full maximum likelihood estimation to ensure complete cases for analysis.

Demographic characteristics showed a gender imbalance consistent with China’s teacher education population: 57 (6.7%) were male students and 789 (93.3%) were female students. Additionally, 241 (28.5%) were freshmen, 235 (27.8%) were second-year students, 299 (35.3%) were juniors, and 71 (8.4%) were seniors. Furthermore, 650 (76.8%) were arts students, 189 (22.5%) were science students, and 9 (1%) chose other fields of study.

### 3.2 Research tools

#### 3.2.1 Professional commitment scale

This study utilized the Professional Commitment Scale for College Students developed by Lian et al. (2005). The scale comprises 27 questions divided into 4 dimensions: Affective commitment (9 items) which assesses emotional attachment to the profession, Ideal commitment (7 items) which evaluates alignment with professional values, Normative commitment (5 items) which measures sense of obligation, and Continuance commitment (6 items) which assesses perceived costs of leaving. All items used a 5-point Likert scale for scoring, with 1 indicating complete non-compliance and 5 indicating complete compliance. The overall alpha coefficient of the scale was 0.9, and the alpha coefficients of the subscales ranged from 0.676 to 0.843. In this study, the alpha coefficients for the subscales of the scale ranged from 0.904 to 0.956.

#### 3.2.2 Learning engagement scale

In this study, I used the learning engagement scale (UWES-S) (the Utrecht Work Engagement Scale-student), which was revised by Li and Huang (2010). The scale consists of 17 questions divided into three dimensions: Motivation (5 items), Vigor (6 items), and Absorption (5 items). In this context, motivation refers to an individual’s enjoyment and interest in learning, understanding the significance of learning, and experiencing happiness in the process; vigor refers to having abundant energy and mental toughness, not getting easily tired from working hard for one’s own learning, and being able to persevere in the face of difficulties; absorption means paying full attention to one’s own learning, being fully engaged, and achieving a state of focus. The scale uses a Likert 7-point scale, with 1 indicating “never” and 7 indicating “very frequently.” A higher total score indicates a higher degree of apparent learning engagement. The alpha coefficients of the revised subscales were found to be between 0.815–0.857, and the alpha coefficient of the total scale was 0.919. In this particular study, the alpha coefficients of the subscales ranged from 0.960–0.961.

#### 3.2.3 Dual system scale for self-control

The study used the revised Adolescent Self-Control Dual-Systems Scale developed by Xie et al. (2014). This scale comprises 21 questions categorized into two systems. They are the Impulsive system (12 items) which include impulsivity (6 items), susceptibility to distraction (3 items), and low delayed gratification (3 items); and the Control system (9 items) which include problem-solving (6 items) and future time perspective (3 items). Responses were rated on a Likert 5-point scale, with 1 indicating non-compliance and 5 indicating high compliance. The alpha coefficient of the revised total scale was 0.82. The alpha coefficients of the subscales in this study ranged from 0.835 to 0.934.

#### 3.2.4 Core self-evaluation scale

The Core Self-Evaluation Scale (CSES) revised by Du et al. (2012) was used. It consists of 10 questions with a unidimensional structure and a 5-point Likert scale. The scale ranges from 1 to 5, with 1 representing complete agreement and 5 representing complete disagreement. The scale also includes some reverse-scoring items. The total score ranges from 10 to 50, with higher scores indicating a higher level of core self-evaluation. The alpha coefficient for this scale is 0.83. The alpha coefficient in this study was 0.954.

### 3.3 Methods of analysis

#### 3.3.1 Common method bias test

In this study, the survey was conducted anonymously. To mitigate any potential biases from the survey administration process, some questions were scored in reverse (Zhou and Long, 2004). The collected data underwent Harman’s one-way test, and an unrotated exploratory factor analysis was applied to all the variables. This analysis revealed a total of 10 factors with eigenvalues greater than 1. The largest factor had an ANOVA contribution rate of 14.94%, significantly smaller than the critical value of 40% (Podsakoff et al., 2003). As a result, it is concluded that there is no significant common method bias in this study.

#### 3.3.2 Data processing

The study’s data analysis comprises three main parts: descriptive statistical scores, validation of the measurement model structure, and structural equation modeling analysis. (1) Descriptive statistical analyses were conducted using SPSS to create frequency distributions of demographic data and calculate the means and standard deviations of the study variables. This helped us to understand the concentration trends and degree of dispersion of the data. (2) The structure of the measurement model was validated through reliability analyses, convergent validity, and discriminant validity. (3) Structural equation modeling (PLS-SEM) was employed to assess model fit, test research hypotheses, and examine the moderating effects within the research model. The evaluation of the PLS-SEM model included assessing the measurement model [composite reliability, average variance extracted (AVE), and discriminant validity], the structural model (*R*^2^ values and path coefficients), and goodness-of-fit tests to determine overall model adequacy (Henseler et al., 2016; Hair et al., 2019; Sarstedt et al., 2019).

## 4 Research results

### 4.1 Analysis of demographic data

The majority of the subjects were female, accounting for 93.3% of the 789 trips. Among the participants, there were 241 (28.5%) freshmen, 235 (27.8%) were second-year students, 299 (35.3%) juniors, and 71 (8.4%) seniors. In terms of the field of study, there were 650 (76.8%) liberal arts students, 189 (22.1%) science students, and 9 (1.1%) in other fields, as indicated in Table 1.

**Table 1:** Analysis of demographic data.

Category	Group	Frequency	Percentage
Gender	Male	57	6.7
	Female	789	93.3
Grade	Freshmen	241	28.5
	Second-year	235	27.8
	Juniors	299	35.3
	Seniors	71	8.4
Major	Liberal Arts	650	76.8
	Science	189	22.1
	Others	9	1.1

### 4.2 Measurement model structure validation

#### 4.2.1 Convergent validity of a model

According to the evaluation criteria of Fornell and Larcker (1981), the standards for assessing the validity of a measurement model include: factor loadings greater than 0.7, composite reliability greater than 0.7, average variance extracted (AVE) greater than 0.5, and Cronbach’s alpha greater than 0.7.

Based on the statistical analysis, it was found that the factor loadings for each dimension in this study ranged from 0.724 to 0.943, all of which were greater than 0.7. Additionally, the component reliabilities for all dimensions ranged from 0.837 to 0.961, also greater than 0.7. The average variance extractions ranged from 0.676 to 0.849, all greater than 0.5. Moreover, the Cronbach’s *α* coefficients of the dimensions ranged from 0.835 to 0.960, all exceeding 0.7. Therefore, the results demonstrate that the measurement model in this study exhibits strong convergent validity. For specific data, please refer to Table 2.

**Table 2:** Measurement model convergent validity analysis.

Construct	Items	Factor loading	Cronbach’s alpha	Composite reliability (CR)	Average variance extracted (AVE)
Affective commitment	CN1	0.848	0.904	0.927	0.676
	CN12	0.718			
	CN13	0.911			
	CN14	0.724			
	CN4	0.845			
	CN7	0.869			
Ideal commitment	CN10	0.845	0.936	0.945	0.723
	CN17	0.841			
	CN2	0.847			
	CN21	0.899			
	CN25	0.898			
	CN5	0.821			
	CN9	0.798			
Continuance commitment	CN11	0.831	0.914	0.979	0.690
	CN19	0.792			
	CN24	0.897			
	CN6	0.797			
	CN3	0.833			
	CN8	0.830			
Normative commitment	CN15	0.919	0.956	0.956	0.849
	CN16	0.934			
	CN20	0.914			
	CN23	0.920			
	CN27	0.921			
Core self-evaluation	HX1	0.832	0.954	0.960	0.707
	HX10	0.860			
	HX2	0.808			
	HX3	0.819			
	HX4	0.839			
	HX5	0.820			
	HX6	0.845			
	HX7	0.872			
	HX8	0.872			
	HX9	0.842			
Impulsivity	KZ1	0.855	0.934	0.936	0.754
	KZ2	0.923			
	KZ3	0.895			
	KZ4	0.897			
	KZ5	0.815			
	KZ6	0.817			
Distractibility	KZ7	0.902	0.835	0.843	0.753
	KZ8	0.895			
	KZ9	0.802			
Poor delay of gratification	KZ10	0.878	0.836	0.837	0.755
	KZ11	0.902			
	KZ12	0.824			
Problem solving	KZ13	0.807	0.934	0.935	0.752
	KZ14	0.904			
	KZ15	0.891			
	KZ16	0.897			
	KZ17	0.859			
	KZ18	0.841			
Future time perspective	KZ19	0.895	0.895	0.896	0.826
	KZ20	0.920			
	KZ21	0.911			
Motivation	TR1	0.900	0.959	0.961	0.831
	TR2	0.894			
	TR3	0.909			
	TR5	0.929			
	TR7	0.918			
	TR9	0.919			
Vigor	TR10	0.928	0.960	0.960	0.833
	TR12	0.937			
	TR15	0.904			
	TR17	0.916			
	TR4	0.884			
	TR8	0.904			
Absorption	TR11	0.943	0.961	0.961	0.864
	TR13	0.926			
	TR14	0.917			
	TR16	0.942			
	TR6	0.921			

#### 4.2.2 Discriminant validity of models

This study uses the average variance extracted (AVE) method to test the discriminant validity between reflective constructs. According to the standards of Fornell and Larcker (1981), when the square root of each construct’s AVE value is greater than the correlation coefficients between that construct and other constructs, it indicates sufficient discriminant validity.

The analysis results show that the square root values of the AVE for most constructs in this study are greater than the correlation coefficients, meeting the requirements for discriminant validity. This indicates that there is sufficient distinction between the reflective constructs, and each construct can effectively differentiate between different concepts. Overall, this study has good discriminant validity. For specific data, please refer to Table 3.

**Table 3:** Measurement model discriminant validity analysis.

Construct	Impulsive system	Learning engagement	Affective commitment	Control system	Core self-evaluation	Ideal Commitment	Continuance commitment	Normative commitment
Impulsive system	**0.779**							
Learning engagement	0.159	**0.881**						
Affective commitment	0.142	0.392	**0.822**					
Control system	−0.255	0.166	0.196	**0.803**				
Core self-evaluation	−0.184	−0.176	0.048	0.118	**0.841**			
Ideal commitment	0.073	0.381	0.422	0.370	0.087	**0.850**		
Continuance commitment	0.208	−0.056	−0.217	−0.069	−0.170	−0.254	**0.831**	
Normative commitment	0.062	0.333	0.312	0.332	−0.022	0.490	−0.206	**0.922**

The bold values on the diagonal are the square roots of the Average Variance Extracted (AVE), which must exceed the off-diagonal correlations to support discriminant validity.

### 4.3 Goodness of fit

The Goodness of Fit (GOF) is an overall measure of the fit of the measurement model, calculated as $\mathrm{GOF}=\sqrt{\bar{\mathrm{AVE}}}\;x\;\sqrt{\bar{R^{2}}}$ . According to Vinzi et al. (2010), a GOF value of 0.1 indicates weak fit, 0.25 indicates moderate fit, and 0.36 indicates strong fit. The results of this study show a GOF of 0.320, indicating a moderate fit.

### 4.4 Path analysis for structural modeling

#### 4.4.1 Path analysis of dimensions of professional commitment to the dual system of self-control

Affective commitment to the impulsive system path coefficient is 0.165, the standard deviation is 0.065 (*t*-value 2.540, *p*-value 0.000 < 0.05), so that affective commitment has a significant effect on the impulsive system is established.

Ideal commitment to the impulsive system path coefficient is 0.050, standard deviation is 0.062 (*t*-value of 0.811, *p*-value of 0.417 > 0.05), therefore ideal commitment has a significant effect on the impulsive system is not established.

Continuance commitment to the impulsive system path coefficient is 0.265, standard deviation is 0.051 (*t*-value 5.203, *p*-value 0.000 < 0.05), so continuance commitment to the impulsive system has a significant effect on the impulsive system is established.

Normative commitment to the impulsive system path coefficient is 0.041, standard deviation is 0.055 (*t*-value of 0.739, *p*-value of 0.460 > 0.05), therefore normative commitment has a significant effect on the impulsive system is not established.

Affective commitment to the control system path coefficient is 0.030, and the standard deviation is 0.065 (*t*-value 0.464, *p*-value 0.643 > 0.05), so it is not valid that Affective commitment has a significant effect on the control system.

Ideal commitment to the control system path coefficient is 0.272, and the standard deviation is 0.065 (*t*-value 4.172, *p*-value 0.000 < 0.05), so the ideal commitment on the control system has a significant effect on the control system is established.

Continuance commitment to the control system path coefficient is 0.047, the standard deviation is 0.073 (*t*-value of 0.637, *p*-value of 0.524 > 0.05), therefore continuance commitment has a significant effect on the control system is not established.

Normative commitment to the control system path coefficient is 0.199, and the standard deviation is 0.061 (*t*-value 3.269, *p*-value 0.001 < 0.05), so normative commitment has a significant effect on the control system is established.

#### 4.4.2 Path analysis of the dual system of self-control to learning engagement

Impulsive system to learning engagement path coefficient is 0.179, and the standard deviation is 0.045, (*t*-value 3.950, *p*-value 0.000 < 0.05), so the impulsive system has a significant effect on learning engagement is established.

Control system to learning engagement path coefficient is 0.209, the standard deviation is 0.051 (*t*-value 4.124, *p*-value 0.000 < 0.05), so the control system has a significant effect on learning engagement is established.

**Figure 2:**
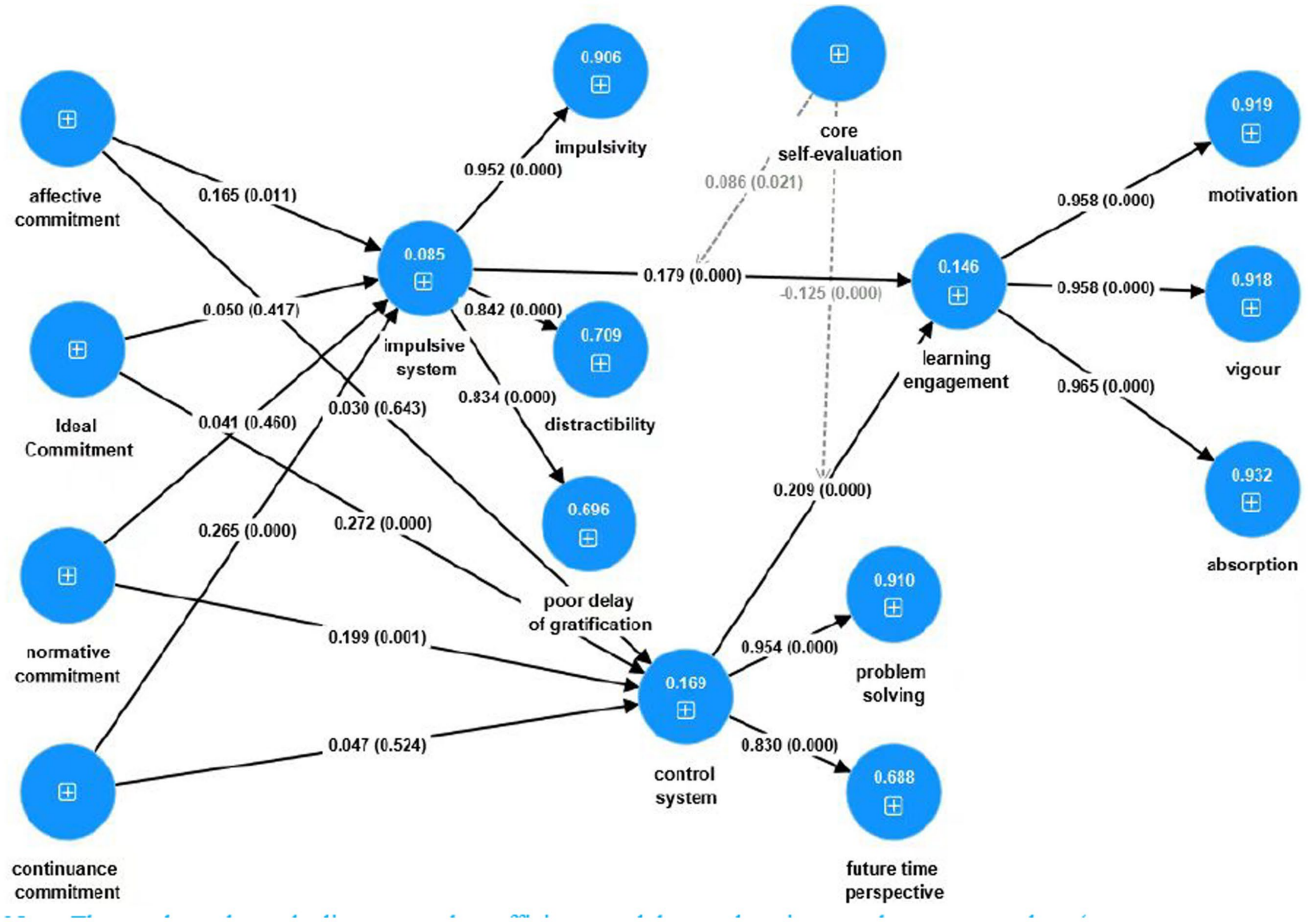
PLS-SEM statistical model diagram. The numbers above the lines are path coefficients, and the numbers in parentheses are *p*-values (*p*-value of *p* ≤ 0.05 is considered statistically significant, *p* ≤ 0.01 highly significant, and *p* ≤ 0.001 very highly significant).

The specific values for the path analysis are shown in the following Table 4 and this result also clearly visible in Figure 2.

**Table 4:** Path analysis.

Path relationship	Path coefficient	Standard deviation	*t*-value	*p*-value
Impulsive system → learning engagement	0.179	0.045	3.950	0.000
Control system → learning engagement	0.209	0.051	4.124	0.000
Affective commitment → impulsive system	0.165	0.065	2.540	0.011
Ideal commitment → impulsive system	0.050	0.062	0.811	0.417
Continuance commitment → impulsive system	0.265	0.051	5.203	0.000
Normative commitment → impulsive system	0.041	0.055	0.739	0.460
Affective commitment → control system	0.030	0.065	0.464	0.643
Ideal commitment → control system	0.272	0.065	4.172	0.000
Continuance commitment → control system	0.047	0.073	0.637	0.524
Normative commitment → control system	0.199	0.061	3.269	0.001

### 4.5 Mediation effects

#### 4.5.1 Analysis of the mediating role of the impulse system

Continuance Commitment → Impulsive System → Learning Engagement, *p* < 0.05, the confidence interval does not include 0 [0.019 to 0.077], indicating that the mediating effect is significant.

Normative Commitment → Impulsive System → Learning Engagement, *p* > 0.05, and the confidence interval includes 0 [−0.013 to 0.028], indicating that the mediating effect is not significant.

Affective Commitment → Impulsive System → Learning Engagement, *p* < 0.05, the confidence interval does not include 0 [0.004 to 0.060], indicating that the mediating effect is significant.

Ideal Commitment → Impulsive System → Learning Engagement, *p* > 0.05, and the confidence interval includes 0 [−0.011 to 0.035], indicating that the mediating effect is not significant.

#### 4.5.2 Analysis of the mediating role of the control system

Ideal Commitment → Control System → Learning Engagement, *p* < 0.05, the confidence interval does not include 0 [0.021 to 0.099], indicating that the mediating effect is significant.

Continuance Commitment → Control System → Learning Engagement, *p* > 0.05, and the confidence interval includes 0 [−0.023 to 0.043], indicates that the mediating effect is not significant.

Normative Commitment → Control System → Learning Engagement, *p* < 0.05, the confidence interval does not include 0 [0.013 to 0.080], indicating that the mediating effect is significant.

Affective Commitment → control system → learning engagement, *p* > 0.05, and a confidence interval includes 0 [−0.018 to 0.038], indicates that the mediating effect is not significant.

Specific data for the analysis of the mediating effect of the dual system of self-control are presented in Table 5.

**Table 5:** Analysis of mediation effect.

Mediation effect	Path coefficient	Standard deviation	*t*-value	*p*-value	2.50%	97.50%
Continuance commitment → impulsive system → learning engagement	0.047	0.015	3.218	0.001	0.019	0.077
Affective commitment → impulsive system → learning engagement	0.030	0.014	2.054	0.040	0.004	0.060
Ideal commitment → impulsive system → learning engagement	0.009	0.012	0.780	0.435	−0.011	0.035
Normative commitment → impulsive system → learning engagement	0.007	0.010	0.714	0.475	−0.013	0.028
Normative commitment → control system → learning engagement	0.041	0.017	2.377	0.017	0.013	0.080
Ideal commitment → control system → learning engagement	0.057	0.020	2.822	0.005	0.021	0.099
Continuance commitment → control system → learning engagement	0.010	0.016	0.600	0.549	−0.023	0.043
Affective commitment → control system → learning engagement	0.006	0.014	0.434	0.664	−0.018	0.038

### 4.6 Moderation effect

Core self-evaluation is the moderating variable, and the data can be seen in Table 6.

**Table 6:** Moderation effect.

Effect	Original sample (O)	Standard deviation (STDEV)	T Statistics (|O/STDEV|)	*P*-values
Core self-evaluation × control system → learning engagement	−0.125	0.034	3.649	0.000
Core self-evaluation × impulsive system → learning engagement	0.086	0.037	2.305	0.021

**Figure 3:**
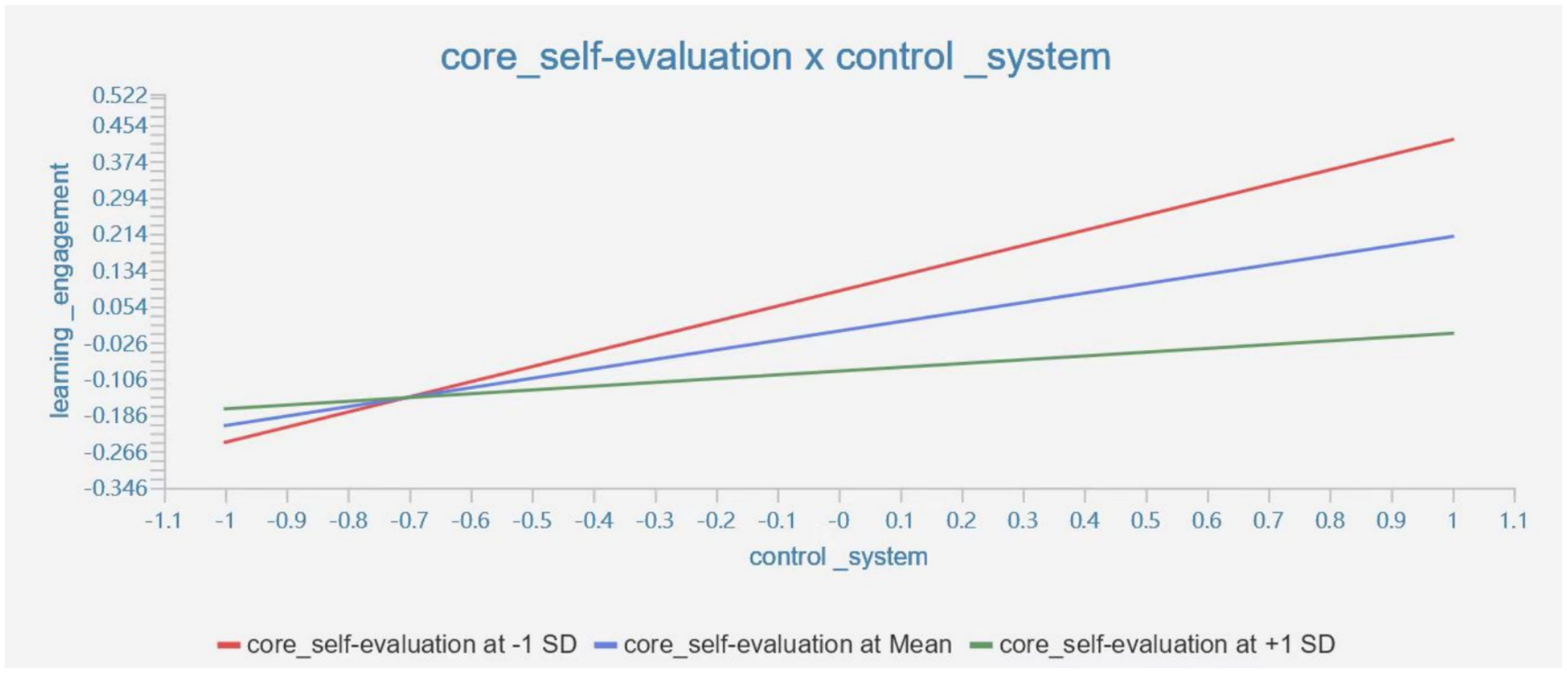
The moderating effect of core self-evaluation × control system on learning engagement.

The moderating effect of core self-evaluation x control system on learning engagement is −0.125 (*T*-value = 3.649 > 1.96, *p* = 0.000 < 0.05), indicating the moderating effect is significant, which can be seen in Figure 3.

**Figure 4:**
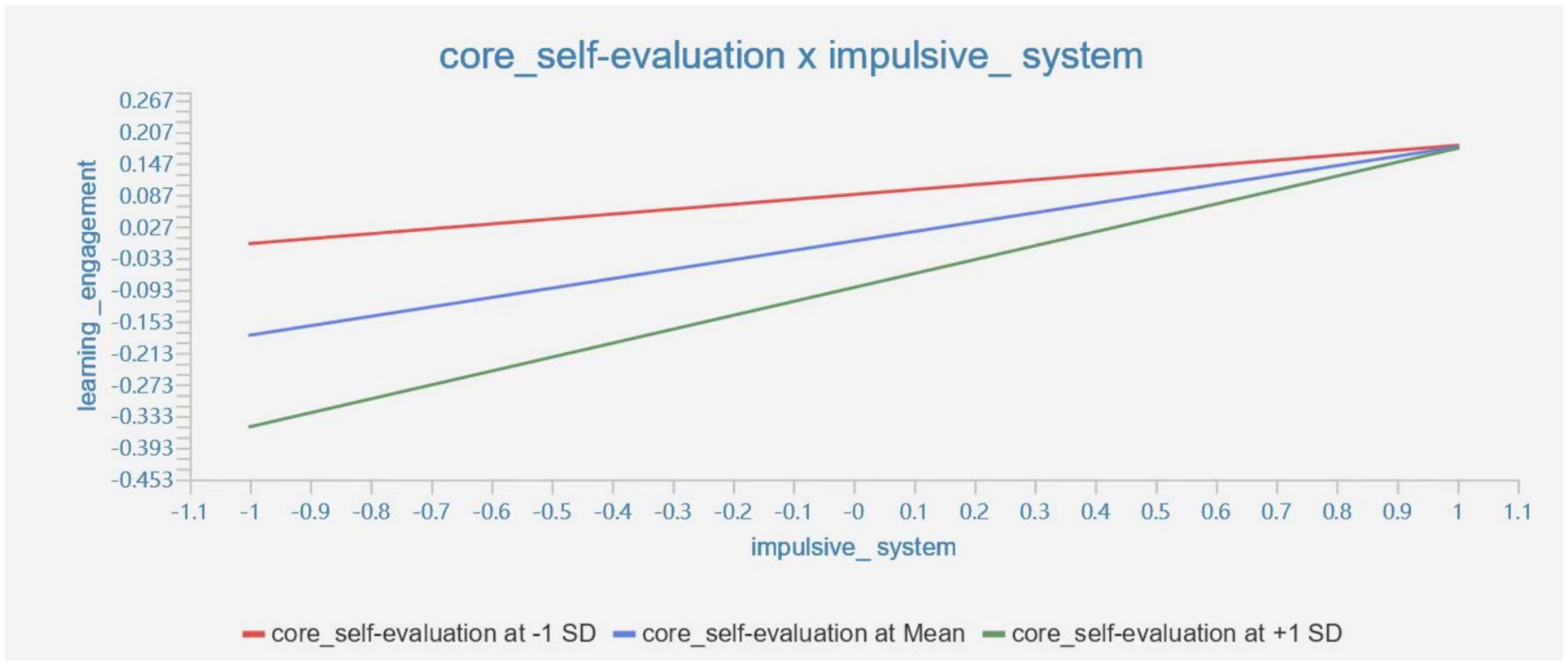
The moderating effect of core self-evaluation × impulsive system on learning engagement.

The moderating effect of core self-evaluation x impulsive system on learning engagement is 0.086 (*T*-value = 2.305 > 1.96, *p* = 0.021 < 0.05), indicating the moderating effect is significant, which can be seen in Figure 4.

## 5 Discussion

Based on the dual-systems theory of self-control, self-determination theory, social cognitive theory, and resource conservation theory, this study examined how the four dimensions of professional commitment among teacher education college students affect learning engagement. The study also investigated the mediating role of self-control and the moderating role of core self-assessment. The findings provided partial support for the research hypotheses.

### 5.1 The impact of four dimensions of professional commitment on the dual system of self-control

The results of the study show that affective commitment and continued commitment significantly positive influence the impulsive system of self-control, this result supports hypotheses H1 and H7; normative commitment and ideal commitment significantly positively influence effect on the control system of self-control, and this result supports hypotheses H4 and H6. The above results are in line with self-determination theory (SDT), suggesting that teacher education students’ professional commitment which rooted in self-will may shapes their self-control behaviors.

Specifically, deep emotional attachment to the teaching profession (affective commitment) and perceived losses upon leaving (continuance commitment) strengthen impulse regulation. Meanwhile, a sense of duty (normative commitment) and alignment with personal ideals (ideal commitment) enhance controlled decision-making. Thus, higher professional commitment corresponds to greater self-control, which helps manage conflicts between commitment and temptation (Kotabe and Hofmann, 2015; Murray et al., 2023). Because of the characteristics of their professional attributes, teacher training college students’ professional commitment to self-control should be of greater concern to educators. Therefore, enhancing affective commitment and continuance commitment of teacher-training college students will help to improve the impulse system of self-control, and enhancing normative commitment and ideal commitment of teacher-training college students will help to enhance the control system of self-control. Thus, given the unique professional demands on teacher-training students, fostering these commitments can improve self-control.

### 5.2 The dual system of self-control has a significant role in learning engagement

The results of the study showed that both the impulse system and control system of self-control significantly positive affect learning engagement. This result supports the research hypotheses H9 and H10. This result is consistent with previous studies, for example, Yang et al. (2024) also concluded that self-control positively affects college students’ learning engagement in their study.

In the current era of networks, college students who lack self-control will inevitably have low levels of learning engagement due to the abundance of network temptations (Antons et al., 2020). However, students with greater self-control are also more able to maintain good study habits, avoid emotional impulses, overcome external environmental temptations, and invest more energy and motivation in their learning engagement (De Ridder et al., 2012).

### 5.3 The mediating role of the dual system of self-control

According to the mediation effect analysis, the impulsive system mediates the positive effects of affective commitment and continuance commitment on learning engagement. Moreover, the control system mediates the positive effects of normative commitment and ideal commitment on learning engagement.

This result is consistent with social cognitive theory, which holds that an individual’s perceptions and ideas guide and shape an individual’s external behavior (Bandura, 1986). In particular, teacher training college students’ affective, continuing, normative, and ideal commitments to the service education profession have an impact on their impulse and control systems of self-control. Their higher professional commitment will increase the level of learning engagement by enhancing individual self-control behaviors.

This mediation emphasize self-control as the bridge between commitment and performance, which is commitment provides the motivation, and self-control converts it into behavioral discipline, ultimately improving engagement. Professional commitment functions as a self-regulatory resource, that is means students with higher commitment to professional values, which suppresses distractions and mobilizes cognitive resources for sustained engagement. This explains why committed students exhibit stronger self-control, translating to better learning engagement.

### 5.4 The moderating role of core self-evaluation

The results of the study showed that core self-evaluation positively moderated the impact of the impulsive system on learning engagement, while negatively moderating the impact of the control system on learning engagement. This result supports the theory of resource conservation (Alarcon et al., 2011). Core self-evaluation is to some extent a protective factor for learning engagement. When the factors that cause the self-control impulse system to function (e.g., cell phone games) reach a certain level, higher levels of core self-evaluation are more protective of learning engagement. The reason for this may be because individuals tend to overestimate their own abilities when they have a high self-evaluation of themselves. They have a stronger sense of control over the factors that influence their impulses and believe that they have the ability to control these factors, which translates into higher levels of learning engagement in their behaviors. At the same time, when the control system of self-control has an impact on learning engagement, students with higher levels of core self-evaluation are prone to overestimate their abilities and instead ignore the impact of the control system (e.g., time perspective) on their learning, thus reducing their learning engagement (Gao et al., 2021).

It can be seen that if college students have too high core self-evaluation, it may negatively affect the effect of the control system of self-control on learning engagement. Therefore, too high or too low core self-evaluation may affect the effect of self-control on learning engagement, and objective core self-evaluation will balance the effects of impulse and control systems of self-control on learning engagement.

### 5.5 Recommendations

As future educators, pre-service teachers’ learning engagement and academic performance will significantly impact educational outcomes and ultimately shape national educational development. This study identifies professional commitment, self-control, and core self-evaluation as key psychological factors influencing learning engagement through distinct mechanisms. Based on these findings, I propose a comprehensive intervention framework, which are differentiated approaches should address various dimensions of professional commitment, such as implementing emotion regulation training for affective and continuance commitment to manage impulsive behaviors, while employing cognitive restructuring interventions for normative and ideal commitment to strengthen regulatory mechanisms and cultivate professional responsibility. Additionally, systematic programs incorporating self-efficacy enhancement and growth mindset cultivation should be developed to improve students’ self-awareness and core self-evaluations. This multi-dimensional approach aims to optimize teacher preparation by developing psychological competencies and behavioral regulation skills.

### 5.6 Research limitations and future research directions

Although there are some findings in this study, there are still some limitations: (1) Due to time and energy constraints, this study employed a cross-sectional design, which may limit definitive causal conclusions given the inherent nature of cross-sectional research. Future longitudinal or experimental designs could better be employed for further studies. (2) This study only explored the moderating effect of core self-assessment on learning engagement, and subsequently, it can incorporate more other possible moderating variables on learning engagement. Additionally, factors such as socioeconomic status and academic stress could confound the observed relationships. Future studies should incorporate these variables to refine the model’s explanatory power. (3) Although this study used a random sampling method, the collected data shows a higher proportion of female participants due to the significant gender imbalance among Chinese normal university students. The predominance of female participants reflects the gender distribution in Chinese teacher-training programs but may limit generalizability. Prior research suggests gender differences in self-control strategies (Wei, 2023). Thus, these findings might not fully apply to male teacher candidates. While gender differences are not the focus of this research, it could potentially be a direction for future studies.

## 6 Conclusion

(1) Affective commitment and continuance commitment demonstrated significant positive effects on the impulsive system of self-control, while normative commitment and ideal commitment showed significant positive effects on the control system of self-control.

(2) Both the impulse and control systems of self-control significantly positive affect learning engagement.

(3) The impulsive system mediated the positive relationships between affective commitment/continuance commitment and learning engagement, whereas the control system mediated the positive relationships between normative commitment/ideal commitment and learning engagement.

(4) The effect of the impulse system and control system of self-control on learning engagement is moderated by core self-evaluation.

## Data Availability

The raw data supporting the conclusions of this article will be made available by the authors, without undue reservation.
